# Coordination nano-space as stage of hydrogen ortho–para conversion

**DOI:** 10.1098/rsos.150006

**Published:** 2015-07-29

**Authors:** Takashi Kosone, Akihiro Hori, Eiji Nishibori, Yoshiki Kubota, Akio Mishima, Masaaki Ohba, Hiroshi Tanaka, Kenichi Kato, Jungeun Kim, José Antonio Real, Susumu Kitagawa, Masaki Takata

**Affiliations:** 1 RIKEN SPring-8 Center, RIKEN, 1-1-1 Koto, Hyogo 679-5148, Japan; 2 Fukushima Renewable Energy Institute, AIST, Fukushima 963-0215, Japan; 3 Institute for Integrated Cell-Material Sciences (iCeMS), Kyoto University, Kyoto 606-8501, Japan; 4 Faculty of Pure and Applied Sciences, TIMS and CiRfSE, University of Tsukuba, 305-8571, Japan; 5 Department of Physical Science, Osaka Prefecture University, Osaka 599-8531, Japan; 6 Department of Chemistry, Kyushu University, Fukuoka 812-8581, Japan; 7 Department of Materials Science, Shimane University, Matsue 690-8504, Japan; 8 Japan Synchrotron Radiation Research Institute, SPring-8, Hyogo 679-5198, Japan; 9 Instituto de Ciencia Molecular (ICMol), Universidad de Valencia, 46980 Paterna, Valencia, Spain; 10 Department of Synthetic Chemistry and Biological Chemistry, Kyoto University, Kyoto 615-8510, Japan; 11 Department of Advanced Materials Science, The University of Tokyo, Chiba 277-8561, Japan

**Keywords:** hydrogen storage porous coordination polymer, structure of absorbed H_2_, ortho–para conversion

## Abstract

The ability to design and control properties of nano-sized space in porous coordination polymers (PCPs) would provide us with an ideal stage for fascinating physical and chemical phenomena. We found an interconversion of nuclear-spin isomers for hydrogen molecule H_2_ adsorbed in a Hofmann-type PCP, {Fe(pz)[Pd(CN)_4_]} (pz=pyrazine), by the temperature dependence of Raman spectra. The ortho (o)–para (p) conversion process of H_2_ is forbidden for an isolated molecule. The charge density study using synchrotron radiation X-ray diffraction reveals the electric field generated in coordination nano-space. The present results corroborate similar findings observed on different systems and confirm that o–p conversion can occur on non-magnetic solids and that electric field can induce the catalytic hydrogen o–p conversion.

## 1. Introduction

Hydrogen molecule H_2_ has two different nuclear-spin isomers, para (p) and ortho (o), owing to requirement of symmetry of wave function. Since interconversion between these isomers is a forbidden process in an isolated molecule, it takes a long time constant, 100 h. The o–p conversion accompanied by heat generation causes a boil-off problem in a cryogenic H_2_ storage [1]. Many kinds of o–p conversion catalysis have been studied so far [2–13]. Several models such as the first-order [8] and second-order [9] perturbation models using paramagnetic and/or diamagnetic metals have been theoretically proposed for the o–p conversion catalysis. The conversion process is often facilitated on a solid surface. The distance between H_2_ and atoms on the surface is crucial to reveal the mechanism. The atomic configurations of H_2_ molecules on the surface have recently been visualized by a neutron powder diffraction [14] and scanning tunnelling microscope [15,16]. Here we adopt three-dimensional nano-pore surfaces of porous coordination polymers (PCPs) [17,18] as a platform for the o–p conversion, and report a structural dependence of nuclear-spin states for H_2_ in the Hofmann-type PCP, {Fe(pz)[Pd(CN)_4_]} (pz=pyrazine) [19,20], by a combination of adsorption isotherm, Raman spectroscopy and *in situ* synchrotron radiation X-ray powder diffraction (SR-PXRD). We have successfully confined and converted H_2_ using the nano-space of PCP.

## 2. Material and methods

### 2.1 Synthesis of {Fe(pz)[Pd(CN)_4_]}

The Hofmann-type PCP {Fe(pz)[Pd(CN)_4_]⋅2H_2_O} was synthesized by slow diffusion of the two solutions in a glass tube for more than 3 days at room temperature. First, one solution contained a mixture of (NH_4_)_2_Fe(SO_4_)_2_⋅6H_2_O (0.10 mmol), pyrazine (0.10 mmol) and citric acid 0.2 mmol in 10 ml water was put in a glass tube. Then, K_2_[Pd(CN)_4_] (0.10 mmol) in water (5 ml)/ethanol (5 ml) mixture solvent was carefully poured on the first solution surface. The glass tube was kept undisturbed. Microcrystals suitable for X-ray diffraction were obtained from the interface between two solutions. The guest-free PCP {Fe(pz)[Pd(CN)_4_]} was prepared by heating under vacuum at 423 K for 3 h.

### 2.2 Measurement of hydrogen gas adsorption isotherm

Hydrogen gas adsorption isotherm was measured with an automatic volumetric adsorption apparatus BELSORP-MAX (BEL Japan, Inc.) in an error of ±0.25%, which is caused by resolution of the pressure gauge. Normal hydrogen gas of 6-nine purity was supplied from a gas cylinder for isotherm, *in situ* SR-XRPD, *in situ* Raman measurements.

### 2.3 Synchrotron radiation X-ray powder diffraction experiment

The *in situ* SR-XRPD experiment of H_2_ adsorption was performed at SPring-8 BL44B2. The large Debye-Scherrer camera with an imaging plate as a detector was used to measure powder profiles. The brown sample powders were carefully crushed with a micro spatula to obtain the powders with homogeneous grain size. The powder sample was loaded into a boron-silicated glass capillary with 0.4 mm internal diameter. The capillary was mounted on the goniometer head connected to the gas pressure control system. All the powder diffraction profiles were collected with a 0.01° step. The wavelengths of an incident X-ray were 0.511 and 0.5496 Å. These are slightly lower energy of the K-edge of Pd to minimize fluorescence scattering from the sample. We determined the wavelength by using a standard powder sample of CeO_2_ by NIST. The X-ray diffraction intensities were collected from 2.0° to 68.95° in 2*θ* (0.511 Å) and 2.0° to 59.40° in 2*θ* (0.5496 Å), which correspond to *d*>0.451 Å and *d*>0.5496 Å *d*-spacing range. The beam size was 0.5×1.0 mm^2^. The exposure time of all the data was for 12 min at each measurement. The temperature of the sample was controlled with a He open-flow cooling device, Helijet, by Agilent Technologies, Inc.

### 2.4 Raman spectroscopy

*In situ* microscopic Raman spectroscopy of H_2_ gas adsorption was carried out by an iHR320 spectrometer (HORIBA Jobin Yvon Ltd) in the temperature range of 20–77 K under the temperature control condition in the precision, approximately millikelvin by directly coupling this Raman system to a cryostat. The sorption isotherm measurements for H_2_ gas and the H_2_ gas induction in Raman measurements were carried out using an automatic volumetric adsorption apparatus (BELSORP-max; MicrotracBEL) connected to the cryostat. A Cu cell for holding a sample attached to a H_2_ induction probe of BELSORP-max was mounted on the cold head of the cryostat. Raman excitation source which can be generated by a 532 nm diode pumped solid-state laser injects vertically down to two optical quartz windows. One was the top of the cryostat and the other was the top of the cell. A known weight (*ca* 30 mg) of the dried sample was placed into the cell. Then, prior to all measurements, the sample was evacuated again using the degas function of the analyser for 12 h at 393 K. In these *in situ* Raman measurements, the laser power was at most 40 μW prior to the injection of optical windows. The exposure time and the accumulation number were 120 s and six times, respectively.

## 3. Results and discussion

**Figure 1.**
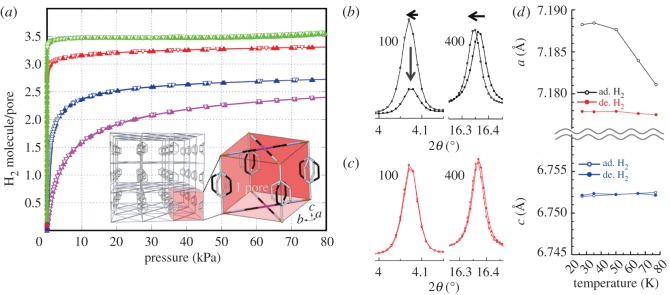
Schematic crystal structure, temperature dependences of adsorption isotherm, lattice constants and Raman spectra of adsorbed H_2_ and {Fe(pz)[Pd(CN)_4_]}. (*a*) Sorption isotherms of H_2_ at 77 K (purple), 65 K (blue), 35 K (red) and 20 K (yellow green). Filled and open triangles show adsorption and desorption processes, respectively. Inset is a crystal structure of three-dimensional porous framework of {Fe(pz)[Pd(CN)_4_]} (left side) and unit pore (right side). 2-D Fe[Pd(CN)_4_] layers extended by Pd–CN–Fe linkages are bridged by pz ligands. (colour code: Fe (orange), Pd (purple), N (light blue), C (black)). (*b*,*c*) Temperature dependences powder diffraction profiles of (*b*) H_2_ adsorbed and (*c*) desorbed states at 35 K (open squares) and 80 K (filled squares). The Intensity of (100) reflection decreased and the positions of (400) reflections shifted with decreasing temperature from 35 to 80 K in the H_2_ adsorbed state. (*d*) Temperature dependences of lattice constants in the H_2_ adsorbed and desorbed states were indicated as open and filled circles, respectively.

Figure 1*a* shows the H_2_ adsorption isotherms at 77, 65, 35 and 20 K. A unit pore volume which corresponds to one unit cell of {Fe(pz)[Pd(CN)_4_]} is shown as an inset. The adsorption amount of H_2_ increased with decreasing temperature. The amounts at 77, 65, 35 and 20 K under 80 kPa are 2.5, 2.7, 3.3 and 3.5 H_2_ per pore, respectively. Approximately one extra H_2_ is adsorbed per pore by lowering the temperature.

Figure 1*b*,*c* shows powder diffraction profiles of (*b*) H_2_ adsorbed and (*c*) desorbed states at 35 K (open squares) and 80 K (filled squares), respectively. All powder profiles show tetragonal symmetry with orientational disorder of the pz pillar ligands. There is a notable different change between the profiles of the H_2_ adsorbed and desorbed states. In the H_2_ desorbed state, peak positions and relative intensities at 80 K are almost identical to those at 35 K indicating no structural change, as shown in figure 1*c*. The lattice constants of the H_2_ adsorbed state showed an anisotropic negative thermal expansion from 80 to 35 K, as shown in figure 1*d*. The expansion is clearly recognized as peak shifts in figure 1*b*.

Accurate structure in charge density level is crucial to understand H_2_ adsorption property of the present system. We optimized an experimental condition to reveal the detailed structure in the H_2_ adsorption process. We measured the data with high counting statistics, approximately 1 000 000 counts, and wide *d*-spacing range, *d*>0.45 Å , at 35 and 65 K in the H_2_ adsorbed and desorbed states, respectively. We determined accurate charge densities, *ρ*(***r***) [21], and electrostatic potentials, *ϕ*(***r***) [22], by combining the maximum entropy method (MEM) and Rietveld refinement [23].

**Figure 2.**
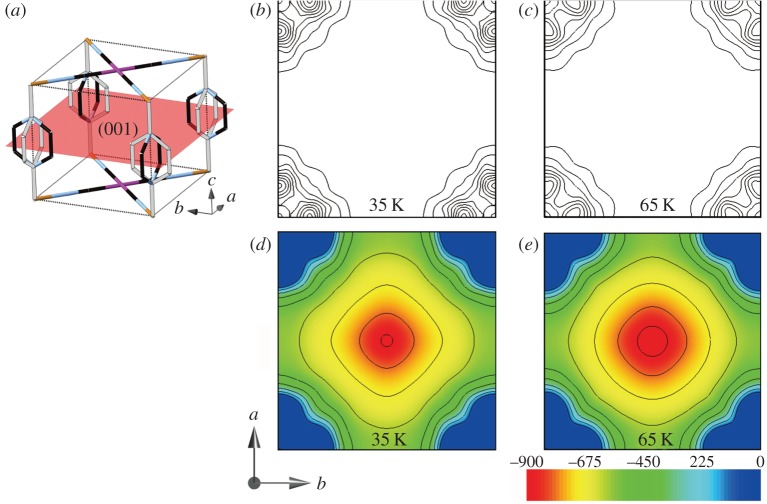
MEM charge densities and electrostatic potentials of {Fe(pz)[Pd(CN)_4_]}. (*a*) The (001) plane in the unit pore. Orange, purple, light blue and black sticks represent the Fe, Pd, N and C atoms, respectively. (*b*,*c*) Contour maps of the charge densities on the (001) plane at (*b*) 35 and (*c*) 65 K. Contour lines are drawn from 0.10 to 1.00 with 0.10 e Å^−3^ intervals. (*d*,*e*) Contour maps of the electrostatic potentials on the (001) plane at (*d*) 35 K and (*e*) 65 K. Contour lines are drawn from −0.90 to 0.00 with 0.10 e Å^−1^ intervals.

**Figure 3.**
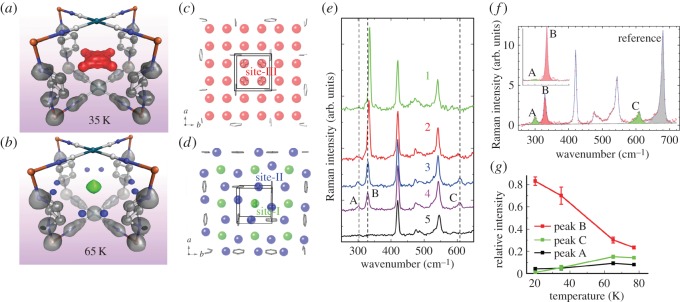
MEM charge densities, structures of H_2_ absorbed in {Fe(pz)[Pd(CN)_4_]}, temperature dependences of Raman spectra, profile fitting result of Raman spectra and temperature dependence of relative peak intensities. (*a*,*b*) MEM charge densities as equi-density contour surfaces in H_2_ adsorption at (*a*) 35 and (*b*) 65 K. The equi-density levels are 1.30 e Å^−3^ for host framework (grey) and 0.20 e Å^−3^ for H_2_ (35 K: red, 65 K: yellow green and blue). (*c*,*d*) Schematic of H_2_ sites for (*c*) site-III and (*d*) sites-I, -II. Green, blue and red spheres represent hydrogen molecules. Disordered pz-pillars are omitted for clarity. (*e*) Raman spectra at 20 (1: yellow green), 35 (2: red), 65 (3: blue) and 77 K (4: purple) in the H_2_ adsorbed states and 77 K (5: black) in the desorbed state as a reference. (*f*) Profile fitting result of Raman spectra at 77 K. The inset shows the result at 20 K in the *x*-range from 260 to 400 cm^−1^. Intensities of peaks A, B and C are filled with yellow green, red and yellow green, respectively. The peak at 680 cm^−1^ which is used as a reference is filled with grey. (*g*) Temperature dependence of relative peak intensities.

Figure 2 shows the *ρ*(***r***) and *ϕ*(***r***) in desorbed state as contour maps on the (001) plane. There is no charge density peaks in the pore space at both 35 and 65 K, indicating no residual gas. Local minima of the *ϕ*(***r***) was found at the pore centre at 35 and 65 K. Figure 3*a*,*b* shows the *ρ*(***r***) in the H_2_ adsorbed states at 35 and 65 K as an isosurface with structure model. There are *ρ*(***r***) peaks showing H_2_ in the pore. As no overlap of the *ρ*(***r***) was observed between the adsorbed H_2_ molecules and framework, H_2_ molecules are physically trapped in the pore. We consider that adsorbed H_2_ behaves as not atom and ion but a molecule. The peak at 65 K was found at the centre of the pore on the (001) plane (a green sphere, figure 3*b*). We also found eight charge density peaks between the pz rings along *a*- and *b*-axes at 65 K (blue spheres, figure 3*b*), whereas the central peak at pore extended to diagonal (a red area, figure 3*a*) and the peaks between the pz rings disappeared at 35 K.

We found three H_2_ sites, what we call sites-I, -II and -III. The site-I is the centre of the pore, 1/2, 1/2, 0; the site-II between the pz rings, 0.41, 0, 0 and site-III is 1/4, 1/4, 0. The schematic structures of H_2_ sites were shown in figure 3*c*,*d* as red (site-III), blue (site-II) and green (site-I) spheres. The charge densities of H_2_ at 65 K correspond to the mixture of the sites-I and -II. The H_2_ densities at 35 K are interpreted as the mixture of the sites-I and -III. The nearest inter-site distances of the sites-I⋯ II and sites-I⋯ III are 3.681 Å, and 2.060 Å, respectively. There should be repulsion between H_2_ molecules owing to the electrostatic interaction and quantum zero-point motion [24,25]. The H_2_⋯ H_2_ distance in the pore has been estimated by a quantum mechanical calculation using a two harmonic potential model [14]. The study suggested that 3.0 Åis about the shortest distance between two H_2_ molecules. In the case of hexagonal H_2_ crystal at 4.2 K, the intermolecular distance is 3.776 Å [26]. These facts provide us a clue to construct possible H_2_ arrangements.

The sites-I and -II can coexist in a pore and the arrangement was achieved at 65 K (figure 3*d*). The maximum amount of H_2_ per pore in this arrangement is three, which is consistent with the adsorbed H_2_ amount of 2.7. Charge densities at site-I were larger than those at site-II. A number of 2.7 H_2_ per pore was achieved by combining fully occupied site-I and partially occupied site-II. Therefore, an increase of H_2_ uptake from 77 to 65 K should occur at site-II.

The main site of H_2_ at 35 K is site-III. The charge densities at sites-I and -II decreased. The charge density at site-II was almost empty. The hydrogen molecules at sites-I and -II moved to site-III from 65 to 35 K. Site exchanges occurred only with cooling from 65 to 35 K. It is impossible to coexist sites-I, -II and -III in one pore, since the inter-site distances are too short for H_2_⋅⋅⋅H_2_. In addition, site-II cannot coexist with site-III of an adjoining occupied pore because of the too short H_2_⋯H_2_ distance (2.43 Å). The *ρ*(***r***) can be interpreted as mixture of two structures for pores. In many of the pores, H_2_ occupied site-III. The maximum amount of H_2_ per pore for site-III is four. In the rest of the pores, H_2_ mainly occupied site-I. Approximately 75% of H_2_ was located at site-III at 35 K from charge densities and adsorption isotherms. It can be easily calculated by an equation: 4*x*+(1−*x*)=3.3, *x*∼0.76, where *x* is the occupancy at site-III.

Lattice expansion with the H_2_ uptake was mainly caused by the intermolecular repulsion between H_2_ at site-III. The H_2_⋯H_2_ distance of 3.599 Å in site-III is smaller than that in the hexagonal H_2_ crystal. The H_2_ molecules should give and receive intermolecular repulsions. A direction of resultant repulsion force is [110] which should expand *a*- and *b*-axes. There is no expansion of the *c*-axis indicating that four H_2_ molecules were located on the (001) plane without a shift along the *c*-axis. The Fe–N_CN_ bond length on the (002) plane was elongated in the lattice expansion, while the change of Pd-C was within errors.

We observed structural change of the H_2_ arrangement by SR-PXRD and investigated nuclear-spin states of the adsorbed H_2_ by Raman spectra. Temperature dependence of Raman spectra in the H_2_ adsorbed state are shown in figure 3*e*. In the desorbed state at 77 K (spectrum 5; black), Raman bands at 420, 470, 485, 545 cm^−1^ were observed and were assigned the vibrational motions of host framework. Three Raman-active bands were newly appeared at 307 (peak A), 328 (peak B) and 609 cm^−1^ (peak C) and were assigned to the adsorbed H_2_ due to the gas dosing in the same temperature of 77 K (spectrum 4; purple). We found some changes in the intensity and peak position for the Raman bands with cooling from 77 to 20 K. Since selection rules derived from intermolecular interactions, the rotational quantum number *J*, the coupling of nuclear-spin and angular momentum, and the arrangement of H_2_, Raman excitation bands contain a great deal of information. In this study, the Raman excitations corresponding to rotational quanta can be observed. The rotational quanta of H_2_ is given by *S*_0_(*J*)=*E*_0,*J*+2_−*E*_0,*J*_ where *E*_0,*J*_ is the rotational energy in the vibrational ground state (*v*=0; *v* is the vibrational quantum number). The quanta of H_2_ have been investigated both theoretically and experimentally [3]. It is reported that at the low temperature, only the *J*=0, *S*_0_(0)≈354 cm^−1^, and *J*=1, *S*_0_(1)≈587 cm^−1^, levels are populated and assigned to p- and o-H_2_, respectively. We observed two peaks A and B around 300 cm^−1^ corresponding to the *S*_0_(0) spectra of p-H_2_ and a broad peak C around 600 cm^−1^, corresponding to the *S*_0_(1) spectra of o-H_2_. The peaks A and C decreased with cooling and peak B shifted and increased at 35 K. Relative intensities of the peaks A, B and C were estimated by the profile fittings as shown in figure 3*f*. Figure 3*g* shows temperature dependence of relative intensities of peaks A, B and C. The intensities were normalized by the intensity of a peak from host framework at 680 cm^−1^. The position of peak B changed from 328(1) cm^−1^ at 77 and 65 K to 333(1) cm^−1^ at 35 and 20 K.

Temperature dependences of the Raman intensities for A and C were very similar indicating the same site occupation of nuclear isomers in figure 3*g*. Peak A was the most red-shifted from the gas phase values. The electrostatic potential around site-I was almost spherical as shown in figure 2*d*,*e*. Site-I is only one minima of the electrostatic potential in the pore. There can be the highest interaction between the adsorbed H_2_ and the host framework at site-I. Therefore, peak A was assigned to p-H_2_ at site-I, and peak C was also assigned to o-H_2_ at site-I. Site-I was occupied by a mixture of o- and p-H_2_ at 77 and 65 K. The ratio of o- to p-H_2_ from Raman intensities at site-I is approximately 3:2 which is the midpoint between equilibrium ratios at 77 K (1:1) and at 300 K (3:1) of normal H_2_. Relative peak intensities of A and C did not change from 77 to 65 K in figure 3*g*. This is also consistent with a full occupation of site-I at 77 and 65 K.

Peak B at 35 K was easily assigned to site-III from Raman spectra and the structure. Therefore, peak B at 77 and 65 K can be assigned to p-H_2_ at site-II. Relative intensities of peak B increased from 77 to 65 K consistent with a partial occupation of site-II. In addition, peak B did not shift from 77 to 65 K which also support our assignment of Raman peaks.

The peak broadening and shift similar to peak B was recently observed in the Raman study of H_2_ in ZnO [27]. In this case, the Raman bands of framework changed with cooling and the shift was interpreted by the interaction between rotational mode of confined H_2_ (at around 320 cm^−1^) and the framework phonons (at 379 cm^−1^). In our case, Raman bands of framework were not observed in the vicinity of peak B and also showed no change. Therefore, the shift of peak B was assigned to the migration of H_2_ between site-II and site-III from the experimentally determined structures.

The Raman peaks around 600 cm^−1^ had almost disappeared within experimental noises at 35 and 20 K indicating that o-H_2_ was converted to p-H_2_. The intensity ratio between peak C and a sum of the peaks A and B from profile fittings was less than 7(3)% at 35 K and 1.2(9)% at 20 K. The result supports that most of the H_2_ at sites-II and -III are p-H_2_. We propose two principal processes to induce the conversion from o-H_2_ to p-H_2_ in the present PCP: (i) trapping of H_2_ at sites-II and -III, and (ii) site exchange from site-I and -II to site-III by cooling.

**Figure 4.**
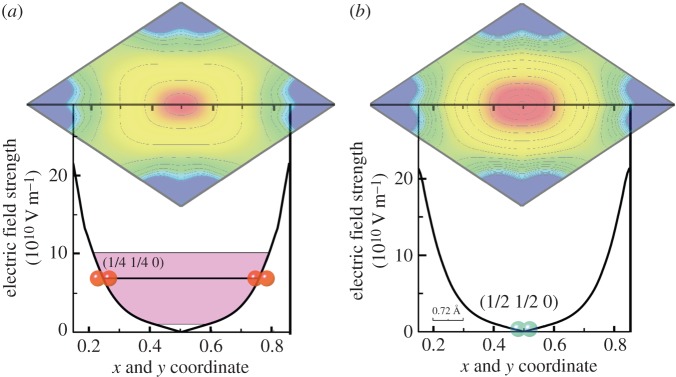
Electric fields at hydrogen sites. (*a*,*b*) One-dimensional absolute electric fields, |*E*(***r***)|, in desorbed state at (*a*) 35 and (*b*) 65 K are plotted along [110] direction on the (001) plane. Blue and red diatomic spheres represent the location of the sites-I and -III, respectively. The purple masking in figure 4*b* shows the range of ***E***(***r***)=10^10^−10^11^ Vm^−1^.

We investigated structural difference between sites-I, -II and -III using the charge densities and electrostatic potentials experimentally determined. The receiving electric fields of H_2_ from the {Fe(pz)[Pd(CN)_4_]} framework can be estimated from electrostatic potential in the desorbed state as shown in figures 2*d*,*e*. We calculated the electric fields, ***E***(***r***), of sites-I, -II and -III by the gradient of the electrostatic potential, ∇*ϕ*(***r***)=−***E***(***r***). Figure 4*a*,*b* shows absolute electric fields, |***E***(***r***)|, along with [110] direction. The ***E***(***r***) for the site-I is 0.0 Vm^−1^ because of the inversion symmetry. The ***E***(***r***) for sites-II and -III are 7.38×10^10^ Vm^−1^ and 7.62×10^10^ Vm^−1^, respectively. There is a huge difference of ***E***(***r***) between site-I and sites-II, -III.

Recently, fast o–p conversions of the order of one or a few minutes have been observed on the H_2_ physisorbed PCP [28] and amorphous solid water [29]. The several mechanisms; spin-orbit-electric dipole-hyperfine contact (SOPY) [29], ion-molecule electron exchanges (XY) and charge transfer [30,31], have also been proposed theoretically for these fast o–p conversions of non-magnetic insulating catalysts. Since measurement time of the present SR-PXRD and Raman spectrum were about 10 min, an upper limit of the o–p conversion time in the present PCP is proposed at 10 min. Most of the recently proposed mechanisms, such as SOPY, XY and charge transfer, can be applied to the present system. For example, the ***E***(***r***) gradient from sites-I to -III can be applied to SOPY. In order to determine the conversion process experimentally, more conversion time resolution is required.

In this study, we observed a correlation between the structure and nuclear-spin state of H_2_ in the three-dimensional Hofmann-type PCP, {Fe(pz)[Pd(CN)_4_]} providing regular coordination nano-space by a combination of the X-ray charge density study and Raman spectrum. Nano-space constructing by PCPs would offer the design of the desirable electric field. The electric field induced processes for such catalytic hydrogen o–p conversion were proposed using the electric field obtained from X-ray diffraction data. X-ray diffraction and Raman spectra of a bulk powder sample provide us with quantitative time and space averaged information and demonstrate availability of the coordination nano-space as a stage for H_2_ o–p conversion. There are still other possible mechanisms for the o–p conversion such as magnetic impurity effects. In this case, mixing of residual paramagnetic high-spin state was estimated to be less than 1.4%. Quantitative information in one molecule size scale with high time resolution is highly desired in the future. Recent advanced X-ray sources such as X-ray-free electron laser would enable us to access such a study.

## Supplementary Material

This zipped folder contains the supplementaly informantion (PDF) and raw data of SR powder diffraction and Raman spectrum. The raw data are organised into a folder structure with README.txt files used to provide guidance on the contents.

## Data accessibility

The datasets supporting this article have been uploaded as part of the electronic supplementary material. The material contains all powder diffraction data, Raman spectra and adsorption isotherm data used in this study.

## References

[1] Amos WA 1998 Costs of storing and transporting hydrogen. Report of National Renewable Energy Laboratory NREL/TP-570-25106.

[2] Farkas L, Sandler L 1940 On the heterogeneous ortho–para conversion on paramagnetic crystals. J. Chem. Phys. 8, 248–251. (doi:10.1063/1.1750638)

[3] Weitzel DH, Loebenstein WV, Draper JW, Park OE 1958 Ortho–para catalysis in liquid-hydrogen production. J. Res. Natl Bur. Std. 60, 221–227. (doi:10.6028/jres.060.026)

[4] Smauch GE, Singleton AH 1964 Technical aspects of ortho–para hydrogen conversion. Ind. Eng. Chem. 56, 20–31. (doi:10.1021/ie50653a003)

[5] Avouris P, Schmeisser D, Demuth JE 1982 Observation of rotational excitations of H_2_ adsorbed on Ag surfaces. Phys. Rev. Lett. 48, 199–202. (doi:10.1103/PhysRevLett.48.199)

[6] Andersson S, Harris J 1982 Observation of rotational transitions for H_2_, *D*_2_, and HD adsorbed on Cu(100). Phys. Rev. Lett. 48, 545–548. (doi:10.1103/PhysRevLett.48.545)

[7] Ilisca E, Sugano S 1986 New channel in ortho–para hydrogen conversion. Phys. Rev. Lett. 57, 2590–2593. (doi:10.1103/PhysRevLett.57.2590) 10033806 10.1103/PhysRevLett.57.2590

[8] Yucel S 1989 Theory of ortho–para conversion in hydrogen adsorbed on metal and paramagnetic surfaces at low temperatures. Phys. Rev. B 39, 3104–3115. (doi:10.1103/PhysRevB.39.3104) 10.1103/physrevb.39.3104 9948607

[9] Ilisca E 1991 Ortho–para H_2_ conversion on a cold Ag(111) metal surface. Phys. Rev. Lett. 66, 667–670. (doi:10.1103/PhysRevLett.66.667) 10043868 10.1103/PhysRevLett.66.667

[10] Paris S, Ilisca E 1999 Electron-nucleus resonances and magnetic field accelerations in the ortho–para H_2_ conversion. J. Phys. Chem. A 103, 4964–4968. (doi:10.1021/jp990040t)

[11] Niki K, Kawauchi T, Matsumoto M, Fukutani K, Okano T 2008 Mechanism of the *ortho–para* conversion of hydrogen on Ag surfaces. Phys. Rev. B 77, 201404 (doi:10.1103/PhysRevB.77.201404).

[12] Muhida R, Setiyanto H, Rahman MdM, Diño WA, Nakanishi H, Kasai H, Fukutani K, Okano T 2006 Ortho–para H_2_ conversion on multiple-decked sandwich clusters of M(C_6_H_6_)_2_ (M=Mn, Fe, Co) induced by an inhomogeneity of spin density distribution. Thin Solid Films 509, 223–226. (doi:10.1016/j.tsf.2005.09.182)

[13] FitzGerald SA, Allen K, Landerman P, Hopkins J, Matters J, Myers R 2008 Quantum dynamics of adsorbed H_2_ in the microporous framework MOF-5 analyzed using diffuse reflectance infrared spectroscopy. Phys. Rev. B 77, 224301 (doi:10.1103/PhysRevB.77.224301)

[14] Liu Y, Kabbour H, Brown CM, Neumann DA, Ahn CC 2008 Increasing the density of adsorbed hydrogen with coordinatively unsaturated metal centers in metal-organic frameworks. Langmuir 24, 4772–4777. (doi:10.1021/la703864a) 18366228 10.1021/la703864a

[15] Li S, Yu A, Toledo F, Han Z, Wang H, He HY, Wu R, Ho W 2013 Rotational and vibrational excitations of a hydrogen molecule trapped within a nanocavity of tunable dimension. Phys. Rev. Lett. 111, 146102 (doi:10.1103/PhysRevLett.111.146102) 24138258 10.1103/PhysRevLett.111.146102

[16] Natterer FD, Patthey F, Brune H 2013 Distinction of nuclear spin states with the scanning tunneling microscope. Phys. Rev. Lett. 111, 175303 (doi:10.1103/PhysRevLett.111.175303) 24206501 10.1103/PhysRevLett.111.175303

[17] Kitagawa S, Kitaura R, Noro S 2004 Functional porous coordination polymers. Angew. Chem. Int. Edn. 43, 2334–2375. (doi:10.1002/anie.200300610) 10.1002/anie.200300610 15114565

[18] Li H, Eddaoudi M, O’Keeffe M, Yaghi OM 1999 Design and synthesis of an exceptionally stable and highly porous metal-organic framework. Nature 402, 276–279. (doi:10.1038/46248)

[19] Niel V, Martinez-Agudo JM, Muñz MC, Gaspar AB, Real JA 2001 Cooperative spin crossover behavior in cyanide-bridged Fe(II)-M(II) bimetallic 3D Hofmann-like networks (M=Ni, Pd, and Pt). Inorg. Chem. 40, 3838–3839. (doi:10.1021/ic010259y) 11466039 10.1021/ic010259y

[20] Ohba M *et al.* 2009 Bidirectional chemo-switching of spin state in a microporous framework. Angew. Chem. Int. Ed. Engl. 48, 4767–4771. (doi:10.1002/anie.200806039) 19294711 10.1002/anie.200806039

[21] Sakata M, Sato M 1990 Accurate structure analysis by the maximum-entropy method. Acta Cryst. A46, 263–270. (doi:10.1107/S0108767389012377)

[22] Tanaka H, Kuroiwa Y, Takata M 2006 Electrostatic potential of ferroelectric PbTiO_3_: visualized electron polarization of Pb ion. Phys. Rev. B 74, 172105 (doi:10.1103/PhysRevB.74.172105)

[23] Takata M, Umeda B, Nishibori E, Sakata M, Saito Y, Ohno M 1995 Confirmation by X-ray diffraction of the endohedral nature of the metallofullerene Y@C_82_. Nature 377, 46–49. (doi:10.1038/377046a0)

[24] Bauer W, Lantzsch B, Toennies JP, Walaschewski K 1976 Observation of symmetry undulations in the integral cross section of para-H_2_ (*j* = 0) + para-H_2_ ( *j* = 0), and the determination of the attractive potential. Chem. Phys. 17, 19–26. (doi:10.1016/0301-0104(76)85003-3)

[25] Silvera IF 1980 The solid molecular hydrogens in the condensed phase: fundamentals and static properties. Rev. Mod. Phys. 52, 393–452. (doi:10.1103/RevModPhys.52.393)

[26] Bostanjoglo O, Kleinschmidt R 1967 Crystal structure of hydrogen isotopes. J. Chem. Phys. 46, 2004–2005. (doi:10.1063/1.1840975)

[27] Koch SG, Lavrov EV, Weber J 2014 Towards understanding the hydrogen molecule in ZnO. Phys. Rev. B 90, 205212 (doi:10.1103/PhysRevB.90.205212)

[28] FitzGerald SA, Hopkins J, Burkholder B, Friedman M 2010 Quantum dynamics of adsorbed normal- and para-H_2_, HD, and D_2_ in the microporous framework MOF-74 analyzed using infrared spectroscopy. Phys. Rev. B 81, 104305 (doi:10.1103/PhysRevB.81.104305)

[29] Sugimoto T, Fukutani K 2011 Electric-field-induced nuclear-spin flips mediated by enhanced spin–orbit coupling. Nat. Phys. 7, 307–310. (doi:10.1038/nphys1883)

[30] Ilisca E 2013 Hydrogen conversion on non-magnetic insulating surfaces. Eur. Phys. Lett. 104, 18001 (doi:10.1209/0295-5075/104/1800)

[31] Ilisca E, Ghiglieno F 2014 Electron exchanges in nuclear spin conversion of hydrogen physisorbed on diamagnetic insulators. Eur. Phys. J. B 87, 235 (doi:10.1140/epjb/e2014-50282-2)

