# Supplementary material for: Coordination nano-space as stage of hydrogen ortho–para conversion
Source: R Soc Open Sci. 2015 Jul 29;2(7):150006. doi: 10.1098/rsos.150006 (PMC4632575; doi:10.1098/rsos.150006)
Supplement: This zipped folder contains the supplementaly informantion (PDF) and raw data of SR powder diffraction and Raman spectrum. The raw data are organised into a folder structure with README.txt files used to provide guidance on the contents. [file rsos150006supp1.zip › Suppl_data_and_info/T_Kosone_RSOS_SI_rev1.pdf]

Supporting Information of

“Coordination Nano-Space as Stage of Hydrogen Ortho-Para Conversion“

Takashi Kosone, Akihiro Hori, Eiji Nishibori, Yoshiki Kubota, Akio Mishima,Masaaki Ohba, Hiroshi Tanaka,

Kenichi Kato, Jungeun Kim, José Antonio Real, Susumu Kitagawa, Masaki Takata.

Supplementary Notes

SR-XRPD Experiments.

The in situ SR-XRPD experiment of H_2 gas adsorption was performed at SPring-8 BL44B2[1]. The large Debye-Scherrer camera with an imaging plate as a detector was installed in the beamline. The gas and vapor pressure control system (GVPC) for an in-situ gas adsorption experiment[2] is also available in the beamline. GVPC consists of evacuation pumps, pressure gauges, gas flow controllers and valves. The schematic illustration of gas flow using GVPC was shown in Figure S1. Adsorbed gas is introduced from a gas bottle to GVPC through a stainless steel tube. H_2 gas with a purity of 6N and N_2 gas with a purity of 6N5 were used as an adsorbed and purge gases. Pressure of the dosed gas was controlled by the mass flow controller and was monitored using three different precision of pressure gauges (max. pressure 133 kPa, 1.33 kPa, 13.3 Pa). Powder sample with suited for accurate powder structural studies were selected by removing large grains from synthesized crystals. The powder sample was loaded into a boron-silicate glass capillary with 0.4 mm internal diameter. The capillary was mounted on the sample holder connected to GVPC using a stainless steel tube (partly flexible). Connection between the sample holder and glass capillary was sealed using an O-ring. The temperature of the sample was controlled with a He open-flow cooling device; Helijet (Agilent Technologies, Inc.). Powder sample was loaded with the length of 1.0 mm. The size of collimated beam was 0.5 x 1.0 mm^2. Only around the powder sample was cooled by He gas stream. The horizontal distribution of temperature at the sample position was kept within ca. 1 K. The temperatures of the sample position were calibrated by the SR-XRPD of oxygen gas filled in the glass capillary to verify the solid phase transition[3-5] from α-O_2 to β-O_2 ($T_{\alpha\beta}$= 23.9 K), β-O_2 to γ-O_2 ($T_{\beta\gamma}$ = 43.8 K) and γ-O_2 to liquid ($T_{m.p.}$= 54.4 K).

All the powder diffraction profiles as shown in Figure S2 were collected with a 0.01° step. The wavelength of incident X-ray was 0.511 Å. This is slightly low energy of the Pd K-edge to minimize fluorescence scattering. The wavelength was calibrated using NIST standard sample of CeO_2. First, the sample was heated at 380 K under vacuum to evacuate all solvent and air (in this operation, valves were V1:close, V2:open, V3:open, V4:open, V5:close). Next, sample temperature was controlled to 80 K. And then, we measured powder profile to make sure degassing states (V1:close, V2:open, V3:open, V4:open, V5:close).

The powder profiles of desorption state were measured at 35 K, 50 K, 65 K, 70 K and 80 K. The X-ray diffraction intensities were collected from 2.0° to 69.00° in 2θ, which corresponds to d > 0.451 Å d-spacing resolution. The exposure time of all the data was for 12 min at each measurement.

In in-situ H_2 adsorption SR-XRPD experiment, the hydrogen gas was loaded into the capillary at 10 K by using GVPC (V1:open, V2:open, V3~V5:close). The dosed pressure of H_2 gas was 105 kPa. Then, the powder profiles in H_2 adsorption state were measured at 35 K, 50 K, 65 K, 70 K and 80 K (V1:close, V2:open, V3~V5:close). The X-ray diffraction intensities were collected from 2.0° to 69.25° in 2θ, which corresponds to d > 0.450 Å d-spacing resolution. The exposure time of all the data was for 12 min at each measurement.

We also measured the powder profiles of H_2 adsorption/desorption state at 27K and 77K. The wavelength of incident X-ray was 0.5496 Å. The intensities were collected from 2.0° to 60.00° in 2θ, which corresponds to d > 0.550 Å d-spacing resolution. These powder profiles were also used for the determination of the lattice constant.

Determination of the lattice constants by Rietveld Refinement.

The powder profiles were used for the structure analysis by Rietveld refinement [6]. The structure reported by Ohba et al [7] was used as an initial model. The structural parameters of the framework were refined with bonding distance restraints with 1.35 Å for C(2)-N(2), 0.90 Å for C(2)-H(1), and 1.85 Å for N(2)-H(1). Isotropic thermal parameters were used for all atoms. A riding model was used for the hydrogen atoms of pz molecules. The temperature dependences of the lattice constants were determined by using the whole powder pattern fitting as shown in figures S3 and S4, and Table S1.

Raman spectroscopy

Figure S5(*a*) shows raman spectra of heating and cooling process in H_2 adsorption state. Peak shown in the figure can be assigned to the H_2 band [8]. We measured temperature dependence of the peak by heating and cooling. It was found that there is no hysteresis. Figure S5(*b*) shows rotational raman bands of p-H_2 at 20 K, 35 K, 65 K and 77 K with H_2 adsorption and 77 K without H_2. Black dotted lines indicate the peak tops at 20K and 77K. The band (B) at 329 cm^{-1} is increased and shifted to 335 cm^{-1} with decreasing temperature.

Figure S6 shows the assigned vibrational Raman bands [9-11]. The CN stretch appears around 2200 cm^{-1} as a well-resolved doublet (symmetric and asymmetric stretches). Compared to the corresponding $K_2[Pd(CN)_4]$ compounds, spectra has C≡N bands at 2192 and 2204 cm^{-1}, which are at higher wavenumbers than that of free $[Pd(CN)_4]^{2-}$. (11) This suggests that the CN groups of $[Pd(CN)_4]^{2-}$ act as the terminal (M-CN) to the bridging (M-CN-M') ligands. Internal modes of pyrazine can be assigned [10, 12]. They appear in the middle region mainly between 600 and 1600 cm^{-1}.

Figure S7 shows profile fitting results of the Raman spectra at 20K, 35K, 65K and 77K. Split-type Peason-VII function was used to assumed Raman profiles.

Magnetic measurement.

The temperature-dependence of magnetic susceptibility measurements for the sample in guest-free and H_2 adsorbed states were performed using a Quantum Design MPMS-XL5R SQUID magnetometer in the temperature range 15−330 K in an applied dc field of 1000 Oe. Here we used the same powder crystals used in the SR-XRPD and Raman spectrum measurements. The samples were placed in a glass tube and fixed to the end of the sample transport rod. Degased sample was prepared by vacuuming for 2 hours in the SQUID sample chamber at 400 K. H_2 gas was injected into the glass tube through the sample transport rod with controlling its vapor pressure at 293 K. Figure S8 shows temperature-dependence for magnetic susceptibility of the H_2 absorbed sample in the cooling process. Spin transition was observed at around 290 K clearly. Mixing of paramagnetic high-spin state was estimated to be less than 1.4%.

Sample Preparation.

Pyrazine was purchased from commercial sources (TCI) and used without any further purification. Precursor $K_2[Pd(CN)_4]\cdot 3H_2O$ was prepared by in 30 mL of water, $PdCl_2$ (TANAKA PRECIOUS METALS) (5.00 g, 0.0282 mol) and KCN (nacalai tesque) (8.26 g, 0.127 mol) were solved. The solution was heated until the recrystallization started. Then the solution was kept for one day. The yellow crystal was obtained (yield: 8.55 g (86.4%)).

$$PdCl_2 + 4KCN + 3H_2O \longrightarrow K_2Pd(CN)_4\cdot 3H_2O + 2KCl \qquad \textbf{[S1]}$$

Optimization of data resolution for a detection of H_2.

We describe a quality of powder diffraction data. Since an accuracy of determined structure fully depends on the quality of the data such as counting statistics, peak width, and data resolutions. The charge density of H_2 is much weaker than those of framework. The optimization of experimental conditions is required to detect H_2 accurately. A lack of data resolution is well known as one of the serious problems for X-ray charge density study especially in a macromolecule crystallography [13]. We investigated the resolution dependence of charge density by MEM analysis.

Figure S9 (*a*), (*b*) and (*c*) show the resolution dependence of MEM charge densities by using calculated structure-factor F_{cal}. In figure. S9(*b*) and (*c*), there are the physically meaningless charge densities in black dotted circles. It is impossible to detect H_2 in these cases. We found that d > 0.452Å d-spacing range resolution was required to detect H_2 as shown in figure. S9 (*a*).

We also investigated a charge density of H_2 by quantum chemical calculations [14] as shown in figure. S9 (*d*). It is found that the shape of charge density was almost single ellipsoid. It is also found that the level of charge density is an order of several hundred meÅ^{-3}. We determined experimental setups for accurate charge density study based on above mentioned analysis.

Detection of H_2 charge densities by MEM/Rietveld analysis.

The MEM/Rietveld analysis for model reconstruction has been utilized in wide variety of materials [15]. We applied the techniques for detection of H_2. In the first step of analysis, the Rietveld refinement was carried out using a preliminary structural model of the Fe(pz)[Pd(CN)$_4$] framework without assuming any guest molecules inside the nanopores as shown at left side in figures S10 (*a*) and S11 (*a*). The structure determined by single-crystal X-ray diffraction was used for the model of framework. The fitting results of Rietveld refinement were shown in S12 and Table S2. In the refinement, bonding distance and angle restraints were applied so as to maintain intrinsic molecular forms. The integrated intensities of each reflection were evaluated from the observed diffraction patterns using the result of the Rietveld refinement. Actually, the diffraction intensity of each data point is divided into each reflection with the contribution from each reflection using the proportion of the calculated intensities. As a result, all of the observed intensities were divided into each reflection without exception, and the integrated intensities of each reflection based on the observed data were estimated. The structure factors derived from these integrated intensities were used in the MEM analysis with the phases calculated from the structural model. MEM calculation was carried out using a computer program, ENIGMA [16]. Unit cell was divided into 128×128×128 pixels. In the MEM calculation, the total number of electrons in the unit cell was given as a constraint. The number of electrons for adsorbed gas molecules was included in this calculation. The amount of adsorbed molecules was independently examined by the adsorption isotherm. The obtained MEM charge densities are shown at right side in figures S10 (*b*) and S11 (*b*). The framework structure can be clearly seen. Despite the structural model without assuming H_2 molecules, local peaks of charge density were recognized in each pore. It means that the diffraction intensities firmly contain the information of the adsorbed H_2 molecule.

As the next step in the analysis, the modified structural model by assuming H_2 molecules based on the local peak positions in the pore was applied as a new model to the second Rietveld refinement. The results of the final

Rietveld refinement were shown in figures S3 and S4, and Table S3, which are satisfactory a structure determination. Reliability factors R_{WP} and R_I were improved by adding H_2. The features of the obtained MEM charge densities were consistent with the structural model used in Rietveld refinement at the atomic level and thus it was judged as a final result of this analysis. The determined MEM charge densities with models are shown in figures S10 (*b*) and S11 (*b*) with the structure model.

Crystal Structure of H_2 adsorbed Fe(pz)[Pd(CN)$_4$].

The schematic crystal structures in H_2 adsorbed states were shown in figures S13 (*a*) at 65 K and (*b*) at 35 K. In the both structure have tetragonal symmetry with *P4/mmm* space group. The structure of Fe(pz)[Pd(CN)$_4$] framework are almost identical in both figures. The equatorial positions are occupied by one type tetracyanometalates unit, [Pd(CN)$_4$]. Pd ion has planar coordination geometries with the bidentate cyano substituent moiety. This complex has one independent Fe^{II} ion with an octahedrally coordination by six N atoms. In the spin crossover transition of the present system, the Fe-N coordination is changed [17,18]. The selected bond length between metal ion and N atoms are presented in Table S4.

Adsorbed H_2 are shown as blue, geen and red balls in the figure. The figure clearly shows the distances between metal ions and H_2 at 65 K are different from those at 35 K. The H_2-Fe distances are listed in the Table S5. These distances correspond to the range of physisorption interactions (3 ~ 10 Å). In the low temperature phase, there is one H_2-Fe distance. The distance is almost identical to the shorter distance at 65 K. It is found that the interaction between framework and H_2 increased at 35 K.

Calculated electric fields of the sites-I, II, and III.

We calculated electric fields in the pore from experimental electrostatic potential in the degas phase. In the MEM charge density in H_2 desorption state, there are small peaks of charge densities indicating small amount of residual gas. The amount of residual charges is less than 0.01*e*. We omitted the charges from MEM charge density. Then we calculated electrostatic potential from charge density. An electric field is calculated by the gradient of the electrostatic potential:

$$\mathbf{E}(\boldsymbol{r}) = -\nabla\phi(\boldsymbol{r}). \qquad [\mathbf{S2}]$$

Where $\boldsymbol{r}$ is the position, $\mathbf{E}(\boldsymbol{r})$ the electric field vector at a point $\boldsymbol{r}$, $\phi(\boldsymbol{r})$ the electrostatic potential at a point $\boldsymbol{r}$**,** denotes gradient. We calculated $\phi(\boldsymbol{r})$ of whole crystallographic unit cell by using the MEM charge density and atomic positions from Rietveld refinement. We calculated $\mathbf{E}(\boldsymbol{r})$ in the crystal using above equation. We listed $|\mathbf{E}(\boldsymbol{r})|$ at the site-I, II and III in the main text because of $\mathbf{E}(\boldsymbol{r})$ being vector. figures S14 (*a*) and (*b*) show $\mathbf{E}(\boldsymbol{r})$ in desorption state as contour maps on (001) plane.

Supplementary Reference

1. Kato K, Hirose R, Takemoto M, Ha S, Kim J, Higuchi M, Matsuda R, Kitagawa S, Takata M (2010) The RIKEN Materials Science Beamline at SPring-8: Towards visualization of electrostatic interaction. *AIP Conference Proceedings* 1234(1):875-878.

2. Matsuda R, Kitaura R, Kitagawa S, Kubota Y, Belosludov RV, Kobayashi TC, Sakamoto H, Chiba T, Takata M, Kawazoe Y, Mita Y (2005) Highly controlled acetylene accommodation in a metal–organic microporous material. *Nature* 436(7048):238-241.

3. Barrett CS, Meyer L, wsserman J (1967) Antiferromagnetic and crystal structures of alpha-oxygen. *J Chem Phys* 47(2):592-597.

4. Cox DE, Samuelsen EJ, Beckurts KH (1973) Neutron-diffraction determination of the crystal structure and magnetic form factor of γ-Oxygen. *Phys Rev B* 7(7):3102–3111.

5. Meier RJ, Helmholdt RB (1984) Neutron-diffraction study of α- and β-oxygen. *Phys Rev B* 29(3):1387–1393.

6. Rietveld HM (1969) A profile refinement method for nuclear magnetic structures. *J Appl Crys* 2(Part 2):65-71.

7. Ohba M, Yoneda K, Agustí G, Muñoz MC, Gaspar AB, Real JA, Yamasaki M, Ando H, Nakao Y, Sakaki S, Kitagawa S (2009) Bidirectional chemo-switching of spin state in a microporous framework. *Angew Chem Int Ed Engl* 48(26):4767-4771.

8. Mao H-K, Hemley RJ (1994) Ultrahigh-pressure transitions in solid hydrogen. *Rev Mod Phys* 66(2):671-692.

9. Molnár G, Niel V, Gaspar AB, Real JA, Zwick A, Bousseksou A, McGarvey JJ (2002) Vibrational spectroscopy of cyanide-bridged, iron(II) spin-crossover coordination polymers: estimation of vibrational contributions to the entropy change associated with the spin transition. *J Phys Chem* ***B*** 106(38):9701-9707.

10. Wilson Jr EB (1934) The normal modes and frequencies of vibrational of the regular plane hexiagon model of the benzene molecules. *Phys Rev* 45(10):706-714.

11. Akyuz S, Dempster AB, Morehouse RL, Suzuki S (1973) An infrared and raman spectroscopic study of some metal pyridine tetracyanonickelate complexes. *J Mol Struct* 17(1):105-125.

12. Zarembowitch J, Bokobza-Sebagh L (1976) Apport de la coordination des hétérocycles à la connaissance de leurs spectres i.r. et Raman—I. Spectres de la pyrazine. *Spectrochim. Acta Part A* 32(3):605-615.

13. Hodel A, Kim S-H, Brünger AT (1992) Model bias in macromolecular crystal structures. *Acta Cryst* A48(Part 6):851-858.

14. Frisch MJ, Trucks GW, Schlegel HB, Scuseria GE, Robb MA, Cheeseman JR, Scalmani G, Barone V, Mennucci B, Petersson GA, Nakatsuji H, Caricato M, Li X, Hratchian HP, Izmaylov AF, Bloino J, Zheng G, Sonnenberg JL, Hada M, Ehara M, Toyota K, Fukuda R, Hasegawa J, Ishida M, Nakajima T, Honda Y, Kitao O, Nakai H, Vreven T, Montgomery Jr JA, Peralta JE, Ogliaro F, Bearpark M, Heyd JJ, Brothers E, Kudin KN, Staroverov VN, Kobayashi R, Normand J, Raghavachari K, Rendell A, Burant JC, Iyengar SS, Tomasi J, Cossi M, Rega N, Millam JM, Klene M, Knox JE, Cross JB, Bakken V, Adamo C, Jaramillo J, Gomperts R, Stratmann RE, Yazyev O, Austin AJ, Cammi R, Pomelli C, Ochterski JW, Martin RL, Morokuma K, Zakrzewski VG, Voth GA, Salvador P, Dannenberg JJ, Dapprich S, Daniels AD, Farkas O, Foresman JB, Ortiz JV, Cioslowski J, Fox DJ (2009) Gaussian, Inc., Wallingford CT.

15. Takata M (2008) The MEM/Rietveld method with nano-applications – accurate charge-density studies of nano-structured materials by synchrotron-radiation powder diffraction. *Acta Cryst* A64(Part 1):232–245.

16. Tanaka H, Takata M, Nishibori E, Kato K, Iishi T, Sakata M (2002) *ENIGMA*: maximum-entropy method program package for huge systems. *J Appl Cryst* 35(Part 2):282-286.

17. Real JA, Andrés E, Muñoz MC, Julve M, Granier T, Bousseksou A, Varret F (1995) Spin crossover in a catenane supramolecular system. *Science* 268(5208):265-267.

18. Gütlich P, Garcia Y, Goodwin HA (2000) Spin crossover phenomena in Fe(II) complexes. *Chem Soc Rev* 29(6):419-427.

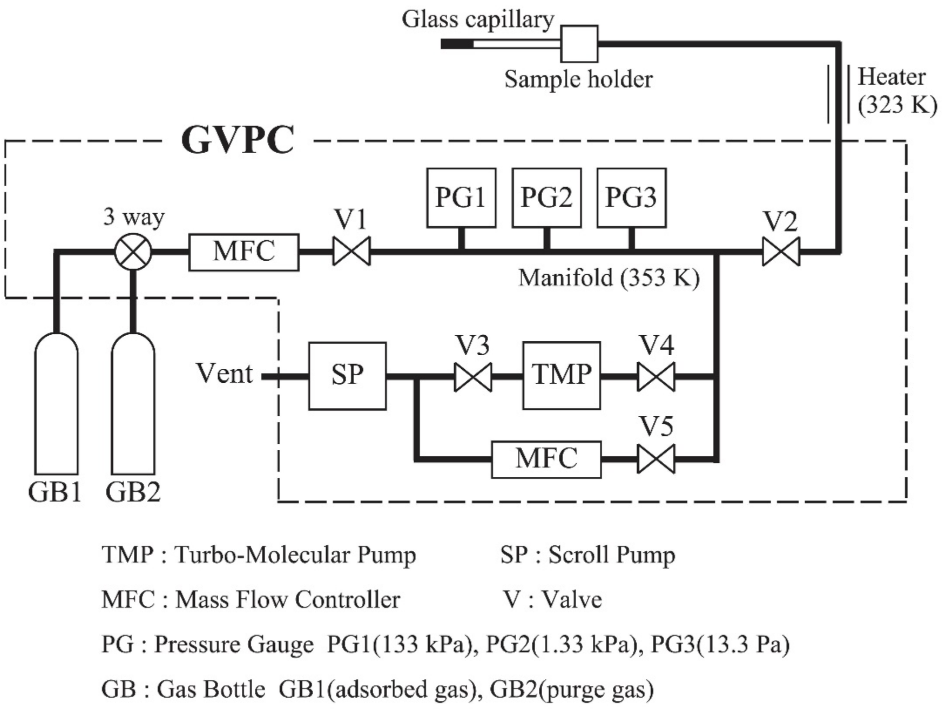

Figire. S1 Schematic illustration of gas flow using GVPC

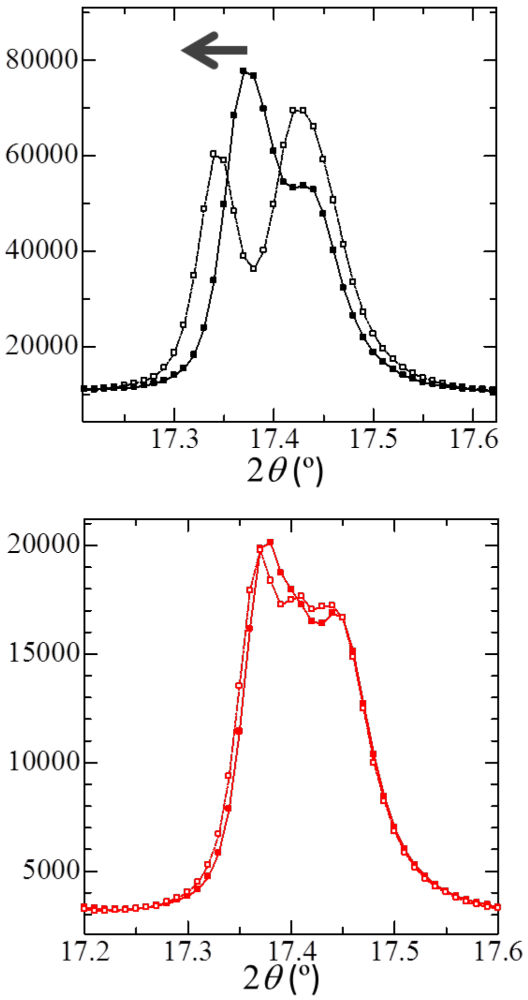

Figure S2. Temperature dependences of powder diffraction profiles at 35 K (open square) and 80 K (filled square) in low and middle angle region. Black and red lines indicate in adsorbed and desorbed H_2, respectively. Black arrows show shifts of the Bragg peaks.

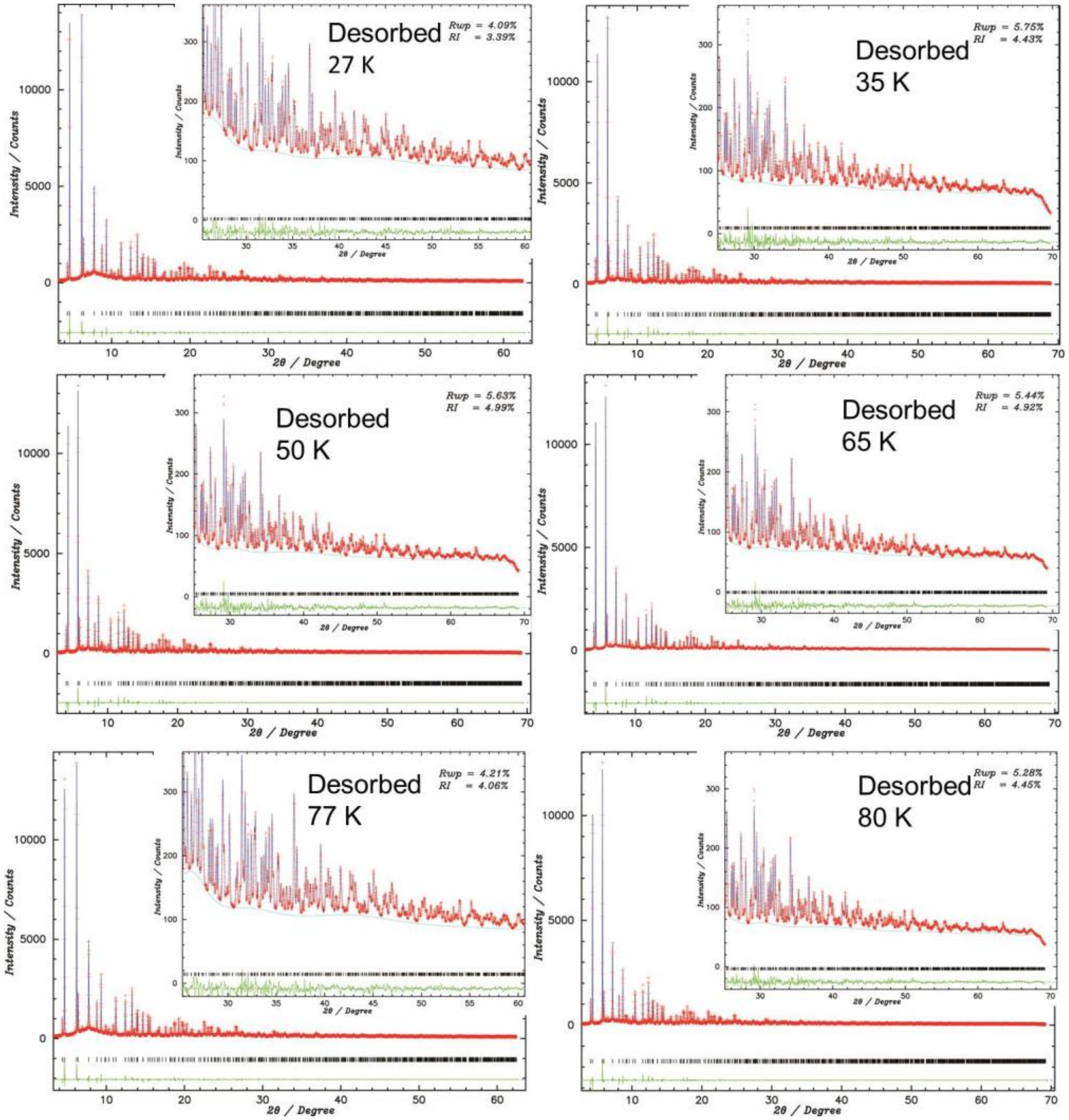

Figure S3. The fitting results of Rietveld analysis. Lattices constants in desorbed state at 27 K, 35 K, 50 K, 65 K, 77 K and 80 K were estimated from the refinement. The insets show the expanded high angle regions. The red crosses and blue lines shows observed and calculated profiles. The green curve is the deviation from observed and calculated profiles. The positions of Bragg reflections are indicated as black ticks.

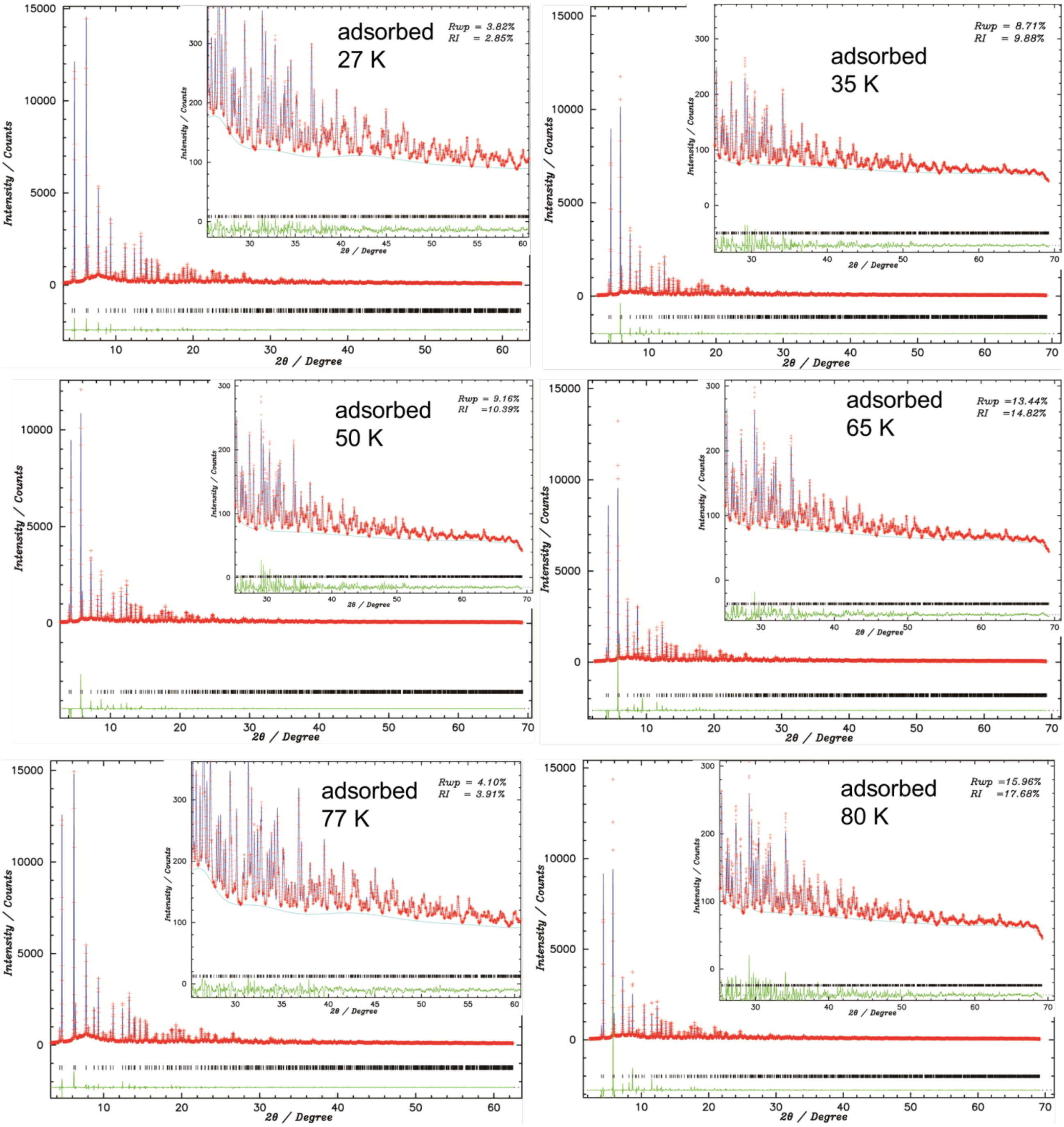

Figure S4. The fitting results of Rietveld analysis. Lattices constants in adsorbed state at 27 K, 35 K, 50 K, 65 K, 77 K and 80 K were estimated from the refinement. The configurations of the figures are same as figure S4.

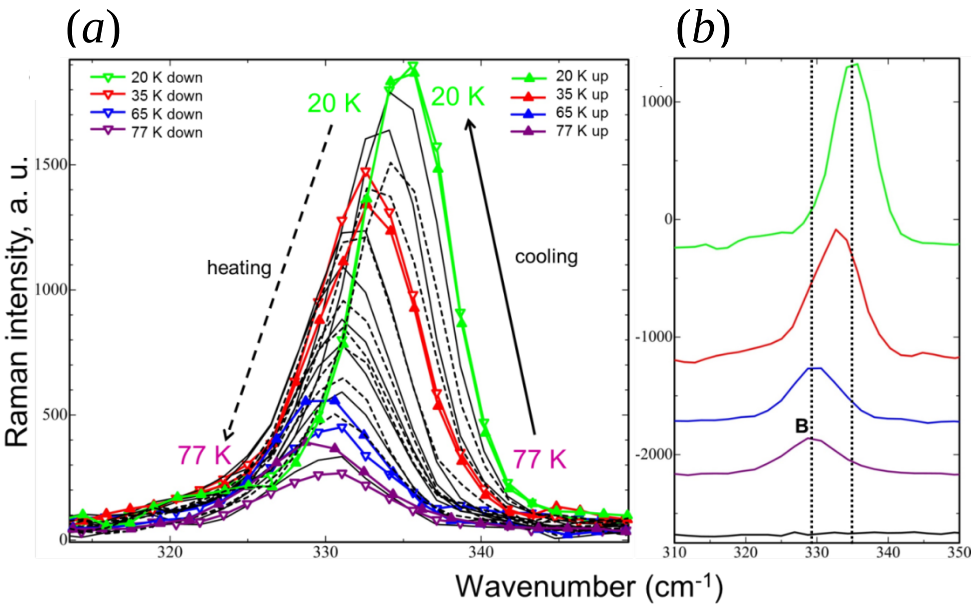

Figure S5. Raman spectra of H_2 adsorbed state at 20 K (green line), 35 K (red line), 65 K (blue line) and 77 K (purple line). (*a*) Black sold and dotted lines are cooling and heating process, respectively. The temperatures at 25 K, 30 K, 40 K, 45 K, 50 K, 55 K, 60 K and 70 K are plotted. Filled and open triangles show cooling and heating processes. (*b*) Raman spectra of adsorbed state with a desorbed state (black solid line). The black vertical dotted lines show the peaks originated from site-I and site-II p-H_2.

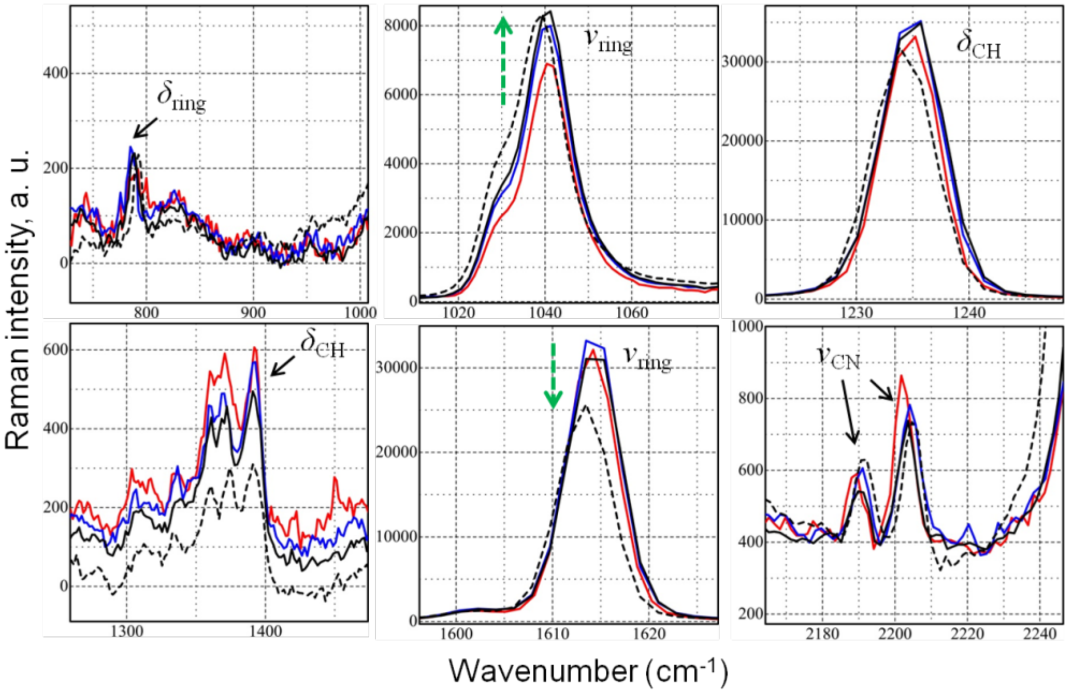

Figure S6. Raman spectra of H_2 adsorbed states at 35 K (red solid lines), 65 K (blue solid lines) and 77 K (black solid lines). Spectra of desorbed state at 77 K are also shown as black dotted lines. Green dotted arrows shows changing intensities. The ν_{CN} is the cyano C≡N stretching mode, the ν_{ring} is the in-plane bending at pz aromatic ring, the δ_{CH} is the in-plane bending at -C-H group of pz plane, and the δ_{ring} is the aromatic ring stretching mode.

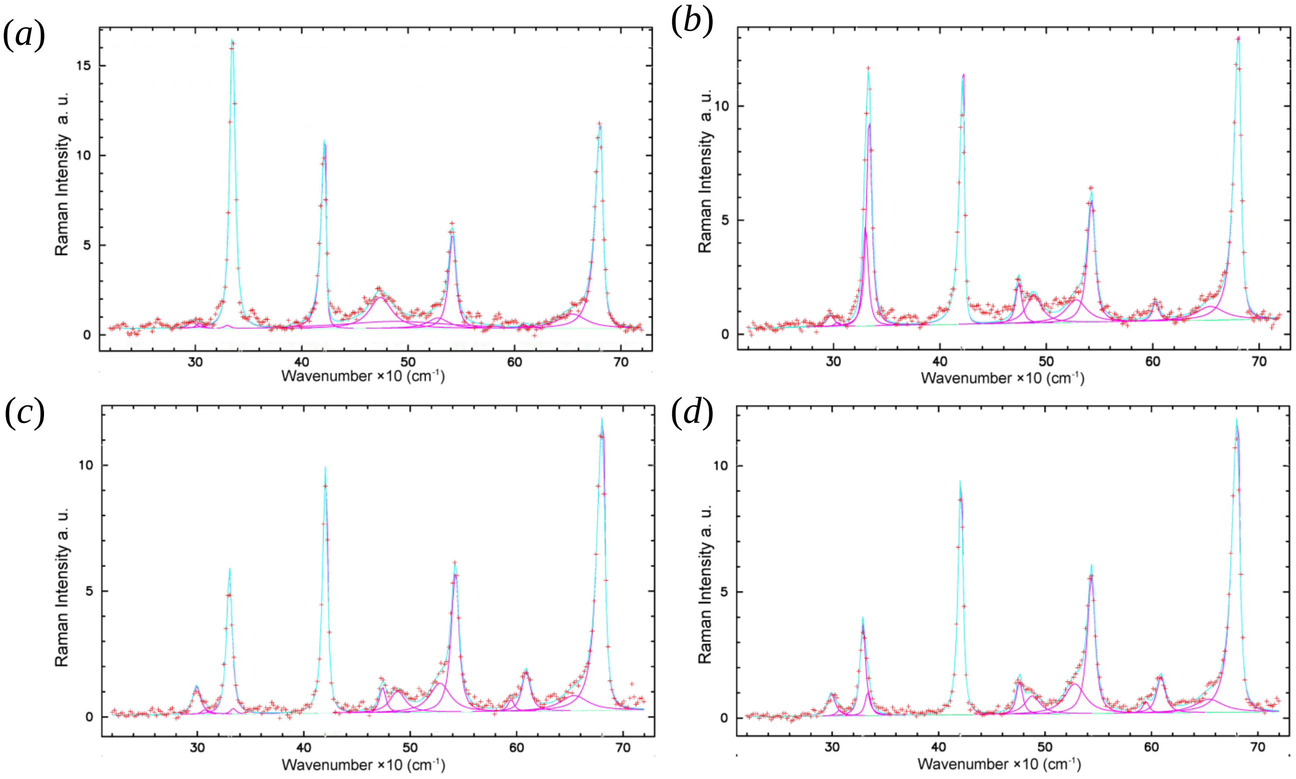

Figure S7. Profile fitting results of Raman spectra at (*a*)20K, (*b*)35K, (*c*)65K and (*d*)77K. Red cross is the observed spectra. Sky blue lines are calculated profiles.

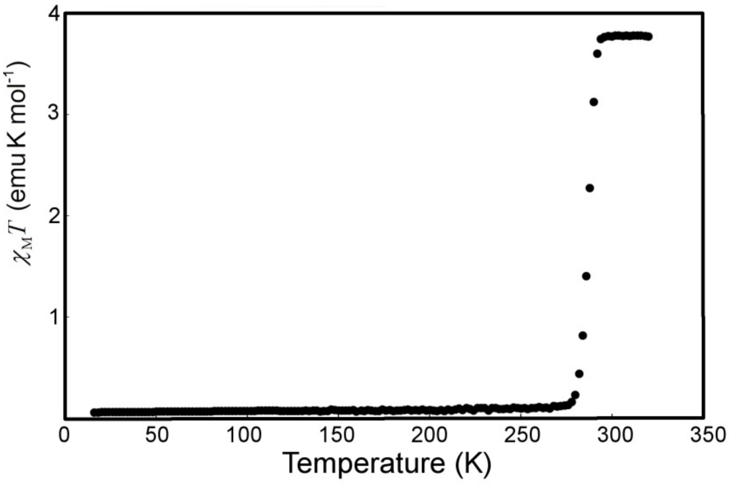

Figure S8. The temperature-dependence of magnetic susceptibility for the H_2 adsorbed sample.

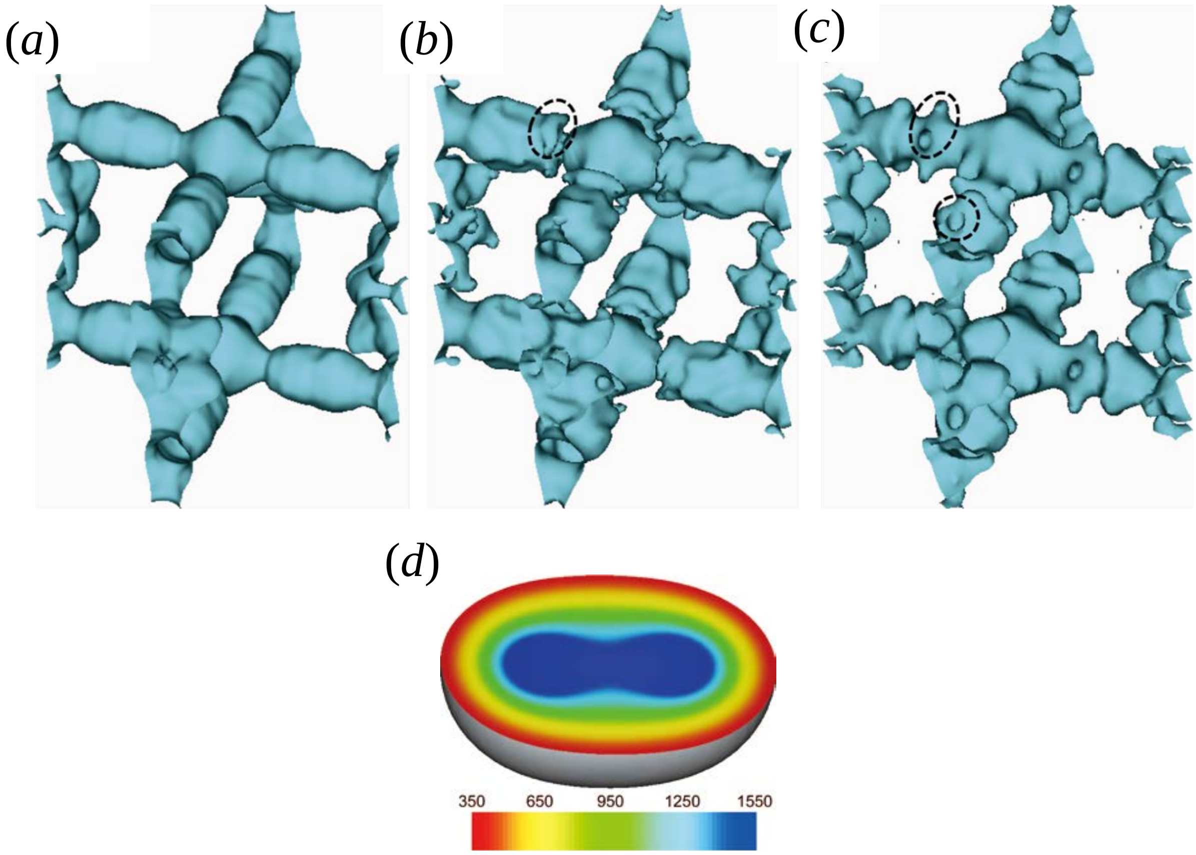

Figure S9. Charge densities calculated to optimize the experimental conditions. **(**a), (b) and (c) Isosurfaces of MEM charge-densities from calculated structure factor F_{cal}, which corresponds to (a) d > 0.452 Å, (b) 0.746 Å and (c) 1.014 Å d-spacing resolutions. The levels of equidensity surface are 500 meÅ^{-3}. (d) The charge density of H_2 as contours and equal density surfaces calculated by Gaussian09 program.[12] Contour lines are drawn from 350 to 1550 at 300 meÅ^{-3} intervals. There are physically meaningless densities peaks in (b) and (c) owing to the Gibbs oscillation from a lack of F_{cal}. These peaks prohibit detecting H_2 since H_2 density is weaker than 500 meÅ^{-3}. No such peaks are observed in (a). We found that the d > 0.452 Å resolution was required to detect absorbed H_2.

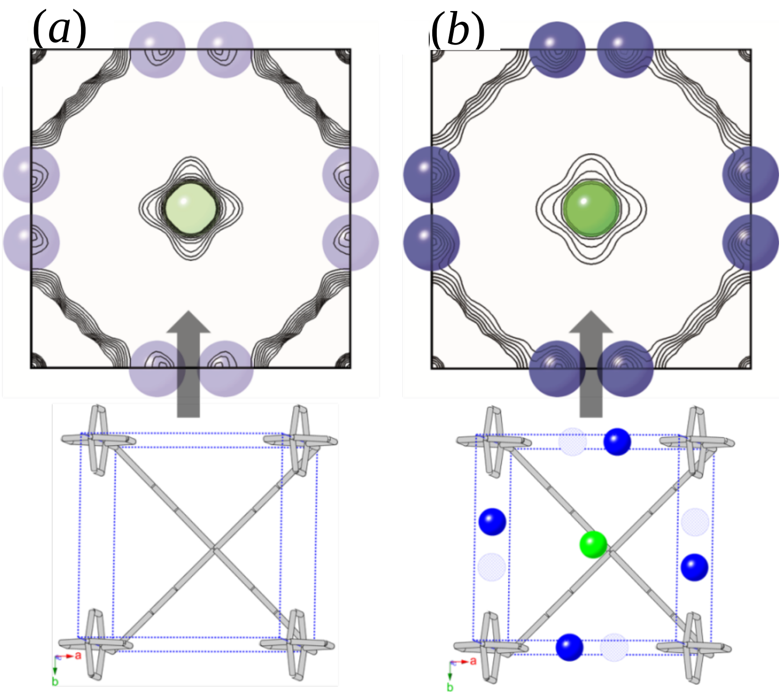

Figure S10. The process of MEM/Rietveld analysis at 65 K. Upper parts show the structure models for Rietveld analysis. Lower parts show the MEM charge density from the observed structure factors obtained in the Rietveld analysis. Contour lines are drawn from 0.12 *e*/ Å^3 to 0.30 *e*/ Å^3 at intervals of 0.03 *e*/ Å^3. (*a*) An initial model and charge density. (*b*) Final model and charge density.

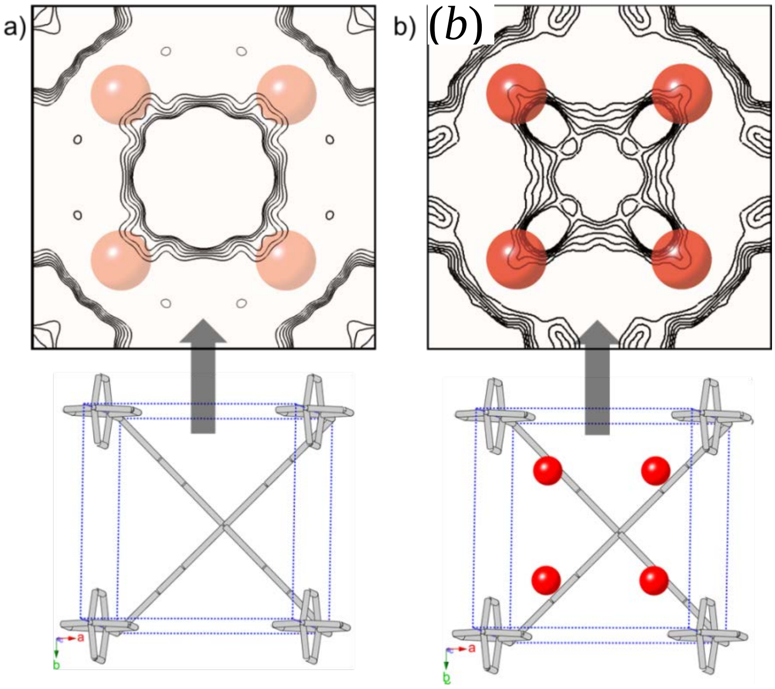

Figure S11. The process of MEM/Rietveld analysis at 35 K. Upper parts show the structure models for Rietveld analysis. Lower parts show the MEM charge density from the observed structure factors obtained in the Rietveld analysis. Contour lines are drawn from 0.12 *e*/ Å^3 to 0.30 *e*/ Å^3 at intervals of 0.03 *e*/ Å^3. (*a*) An initial model and charge density. (*b*) Final model and charge density.

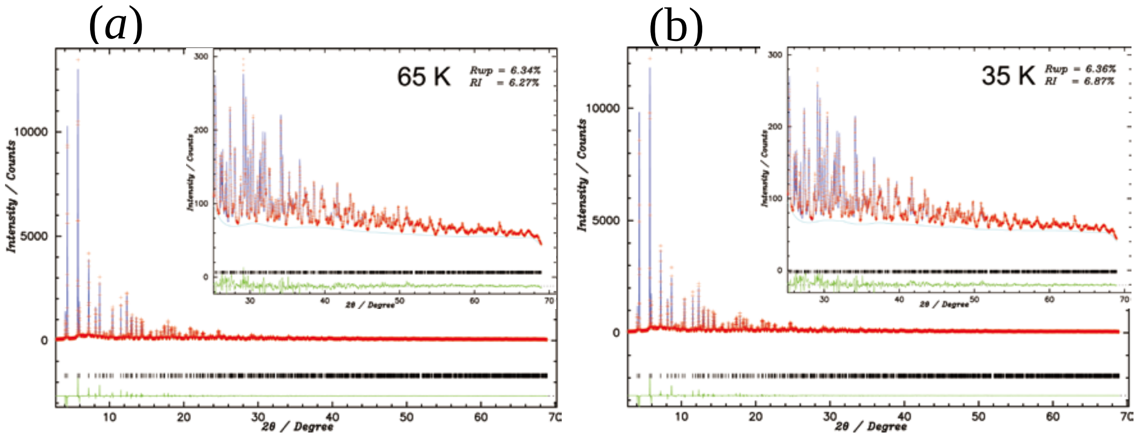

Figure S12. The fitting results of Rietveld analysis by using only the Fe(pz)[Pd(CN)$_4$] framework model at (*a*) 65 K and (*b*) 35 K.

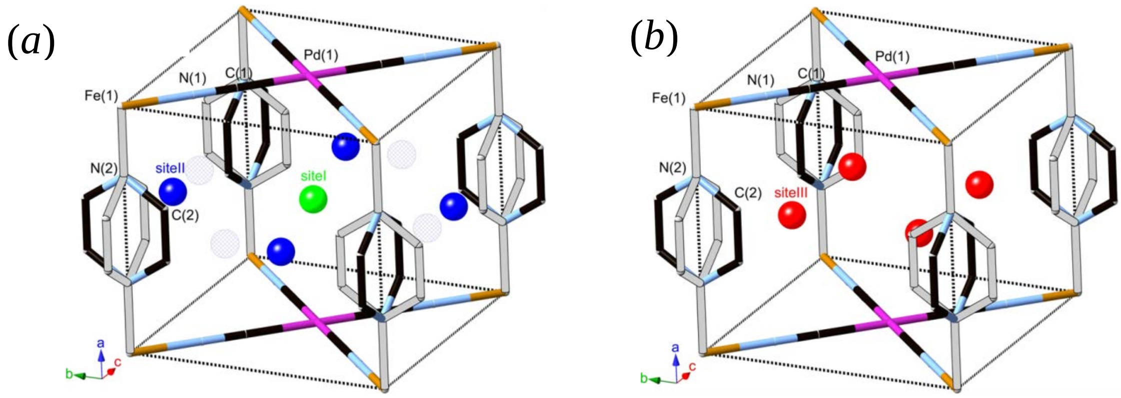

Figure S13. The structures in adsorbed state at (*a*) 65 K and (*b*) 35K. Hydrogen atoms of pz omitted for clarity. The pz ligands are disordered with two orientations. [color code: Fe (brown), Pd (purple), N (light blue), C (black), site-I (green), site-II (blue), site-III (red)] **.**

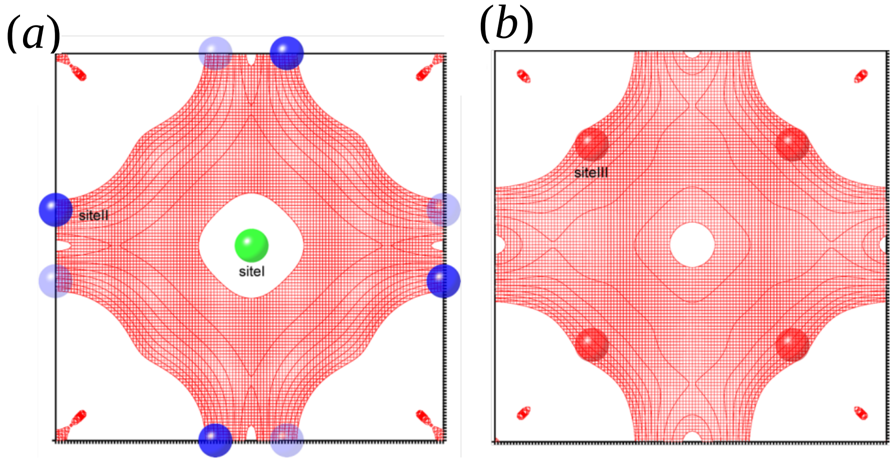

Figure S14. Contour maps of the electric field strength on the (001) plane in desorbed state at (*A*) 65 K and (*B*) 35 K. Contour lines (with red grid) are drawn from 10^{10} to 10^{11} Vm^{-1} at intervals of 2 Vm^{-1}.

Supplementary Tables.

Table S1. Lattice parameters.

**Desorbed state**						
Temperature (K)	80	77	65	50	35	27
Crystal system			tetragonal			
Space group			*P*4/mmm			
a (Å)	7.17719(5)	7.17480(4)	7.17765(3)	7.17791(3)	7.17786(5)	7.17562(4)
c (Å)	6.75261(7)	6.75256(6)	6.75235(5)	6.75224(5)	6.75235(8)	6.75173(6)
V (Å^3)	347.84(1)	347.61(1)	347.87(1)	347.89(1)	347.89(1)	347.64(1)

**Adsorbed state**						
Temperature (K)	80	77	65	50	35	27
Crystal system			tetragonal			
Space group			*P*4/mmm			
a (Å)	7.18136(12)	7.17842(4)	7.18397(10)	7.18767(6)	7.18845(6)	7.18706(4)
c (Å)	6.75536(17)	6.75300(6)	6.75525(14)	6.75518(9)	6.75497(9)	6.74963(6)
V (Å^3)	348.39(1)	347.98(1)	348.63(1)	3478.99(1)	349.05(1)	348.64(1)

Table S2. The analytical details of MEM/Rietved refinement in figure S10.

**Adsorbed state (only host structure model)**		
Temperature (K)	65	35
Crystal system	tetragonal	
Space group	*P*4/mmm	
a (Å)	7.18763(4)	7.18960(6)
c (Å)	6.75145(6)	6.74958(8)
V (Å^3)	348.79(1)	348.89(1)
Reflections	1211	1212
2θ range of analysis	2.50° - 68.95°	2.25° - 68.95°
Final R indices[a], [b]	R_I = 0.063, R_{wp} = 0.064	R_I = 0.069, R_{wp} = 0.064

Table S3. The analytical details of the final MEM/Rietved refinement.

Desorbed state		
Temperature (K)	65	35
Crystal system	tetragonal	
Space group	*P*4/mmm	
a (Å)	7.17713(6)	7.17883(6)
c (Å)	6.75252(7)	6.75297(8)
V (Å^3)	347.83(1)	348.02(1)
Reflections	1209	
2θ range of analysis	2.25° - 69.00°	
Final R indices[a], [b]	R_I = 0.03858,	R_I = 0.03839,
	R_{wp} = 0.05072	R_{wp} = 0.05381

Adsorbed state		
Temperature (K)	65	35
Crystal system	tetragonal	
Space group	*P*4/mmm	
a (Å)	7.18763(4)	7.18960(4)
c (Å)	6.75145(6)	6.74970(6)
V (Å^3)	348.79(1)	348.90
Reflections	1211	1223
2θ range of analysis	2.50° - 68.95°	2.50° - 69.25°
Final R indices[a], [b]	R_I = 0.05584,	R_I = 0.03703,
	R_{wp} = 0.06488	R_{wp} = 0.04720

[a] $R_{wp} = [\Sigma w|y_o - y_c|^2/\Sigma w y_o^2]^{1/2}$, [b] $R_I = \Sigma|I_o - I_c|/\Sigma I_o$.

Table S4. Selected bond length at 35 K and 65 K.

	Adsorbed state		Desorbed state	
Temperature [K]	65	35	65	35
Fe(1)-N-$_{pz}$(1) [Å]	1.928(4)	1.922(3)	1.920(3)	1.926(3)
Fe(1)-N_{CN}(2) [Å]	1.929(4)	1.944(4)	1.940(4)	1.952(4)
Pd(1)-C(1) [Å]	1.996(4)	2.002(4)	1.988(3)	1.989(3)
C(1)-N_{CN}(1) [Å]	1.150(6)	1.152(5)	1.161(4)	1.167(4)

Table S5. Metal…H_2 distances in adsorbed state. I, II and III indicates adsorption sites.

65 K	35 K
M-H_2 [Å]	M-H_2 [Å]
Fe(1)…H_2 (I): 5.075	Fe(1)…H_2 (III): 4.2235
Fe(1)…H_2 (II): 4.18(10)	
Pd(1)…H_2 (I): 3.376	
